# Using stochastic simulation modelling to study occupancy levels of decentralised admission avoidance units in Norway

**DOI:** 10.1080/20476965.2023.2174453

**Published:** 2023-02-15

**Authors:** Meetali Kakad, Martin Utley, Fredrik A. Dahl

**Affiliations:** a Health Services Research Unit, Akershus University Hospital Trust, Lørenskog, Norway; b Institute of Clinical Medicine, University of Oslo, Oslo, Norway; c Clinical Operational Research Unit, Department of Mathematics, University College London, London, UK; d Image Analysis and Earth Observation, Norwegian Computing Centre, Oslo, Norway

**Keywords:** Admission avoidance, community-based, healthcare, discrete event simulation, Erlang, regression

## Abstract

Identifying alternatives to acute hospital admission is a priority for many countries. Over 200 decentralised municipal acute units (MAUs) were established in Norway to divert low-acuity patients away from hospitals. MAUs have faced criticism for low mean occupancy and not relieving pressures on hospitals. We developed a discrete time simulation model of admissions and discharges to MAUs to test scenarios for increasing absolute mean occupancy. We also used the model to estimate the number of patients turned away as historical data was unavailable. Our experiments suggest that mergers alone are unlikely to substantially increase MAU absolute mean occupancy as unmet demand is generally low. However, merging MAUs offers scope for up to 20% reduction in bed capacity, without affecting service provision. Our work has relevance for other admissions avoidance units and provides a method for estimating unconstrained demand for beds in the absence of historical data.

## 1. Introduction

Reducing avoidable admissions to acute hospitals is a priority for many countries. Options for avoiding admissions span preventative measures aimed at reducing acute exacerbations to offering hospital care to patients in their own homes.

Multiple models for community-based admissions avoidance units exist, including nurse-led units, virtual wards, acute step up and/or stepdown units and community hospitals. The evidence is unclear whether these units reduce admissions or readmissions to hospitals (D’souza & Guptha, 2013; Imison et al., 2017). Community hospitals appear to offer at least as good if not better health outcomes as hospitalisation (Garåsen et al., 2008; Imison et al., 2017; Lappegard & Hjortdahl, 2014; Steventon et al., 2018; Young et al., 2007) and may be cost-effective (Garåsen et al., n.d; Lappegard & Hjortdahl, 2012).

The evidence for cost-effectiveness of community-based admissions avoidance units is uncertain, but they are typically popular amongst patients and their families (D’souza & Guptha, 2013; Huntley et al., 2017; Imison et al., 2017; Monitor, 2015; National Audit Office, 2018; The Health Foundation, 2011; The King’s Fund, 2010). Whilst nurse-led care may reduce the number of early readmissions, it has been shown to be more expensive than usual care, largely due to longer length of stay (Griffiths, 2006). Admissions to admission avoidance units may reduce the risk of admission to long-term care by maintaining functional levels and independence amongst otherwise vulnerable elderly patients (Garåsen et al., 2008; Lappegard & Hjortdahl, 2014; Monitor, 2015; Shepperd et al., 2016).

In this study, we focus on a community-based admission avoidance units in Norway. Norway has a highly dispersed population and, like Canada and Australia, faces challenges in providing cost-effective, high-quality specialist care to citizens in more remote settings. Historically, community-based acute care has been used to reduce the dependency on scarce hospital beds in sparsely populated areas (Aaraas et al., 1998, 2000; Lappegard & Hjortdahl, 2013; Leonardsen, Lappegard, et al., 2017). However, in 2016, it became mandatory for *all* municipalities in Norway to establish community-based beds for admission avoidance purposes, known as municipal acute units (MAUs) (Act relating to municipal health and care services, etc. (Health and Care Services Act), 2011 ; Regulations for the Duty of Municipalities to Provide 24 Hour Urgent Care Beds, 2016; Norwegian Ministry of Health and Care Services, 2009). The intention was to reallocate 240,000 acute patient days away from the hospital wards and to municipal settings (2012; Norwegian Directorate of Health, 2011). The beds were intended for use by patients with sub-acute or acute conditions with low likelihood of physiological deterioration who required admission but could be safely managed in a community bed.

As of 2019, 216 MAUs were established in Norway serving 406 municipalities. The majority of units have three beds or less and are often embedded in larger structures such as nursing homes or co-located with other municipal health care emergency services though typically function as independent units (Norwegian Directorate of Health, 2020a). MAUs face issues related to their small size in many municipalities, making them vulnerable to variation in numbers of arrivals, resulting in low absolute mean occupancy levels for which they have received criticism (Bruun Wyller, 2014; Nilsen et al., 2017). There has also been concern as to whether the initial policy goal was based on an unrealistic level of demand. Previous work has shown that small size and relatively low and variable demand for MAU beds results in a paradoxical situation where a greater volume of patients and MAU beds would be required nationally to meet the initial policy goal (assuming current distribution of MAUs), whilst at the same time individual MAUs are criticised for having low occupancy (Kakad et al., 2019). Regression modelling to understand drivers of patient flows through MAUs has also been carried out (Kakad et al., 2020).

A recent study reported that it was difficult to determine the cost-effectiveness of MAU beds as municipalities struggle to attribute specific costs to MAU beds. This may be because resources are shared across municipal functions (Oslo Economics et al., 2019). Islam and Kjerstad tentatively conclude that MAUs could be a useful and potentially cost-effective component in chronic disease management. This is accompanied by a caveat that the cost-effectiveness of MAUs assumes that hospital beds freed up by MAUs are used for patients with more serious illnesses. It also assumes that the quality of care offered at MAUs is equivalent to, if not better than, that offered in hospital (Islam & Kjerstad, 2019).

Resolving low MAU occupancy remains an issue for decisionmakers. Simulation modelling of strategies for increasing MAU occupancy can provide useful information on the impact of organisational changes prior to implementation.

Simulation is considered a useful tool for assessing and evaluating complex problems and systems (Burch, 2018). In particular, simulation models are particularly good at modelling variability within systems, such as the arrival rate of patients to the emergency department (Robinson, 2014a). By using computers to repeatedly imitate real-life processes within a system, simulation models can generate outputs that can be used to estimate system performance or capacity requirements (Law, 2015).

Simulation has been widely used in health care with applications ranging from capacity planning and resource allocation within health care facilities, performance comparisons, scheduling of procedures, and the development of health policy and programs (Bagust et al., 1999; Fialho et al., 2011; Fone et al., 2003; Jun et al., 1999; Pitt et al., 2016; Ramwadhdoebe et al., 2009; Brailsford et al., 2009; Thorwarth & Arisha, 2009). Most simulation studies in healthcare focus on acute care settings and improving efficiencies in hospitals, even though much of healthcare provision occurs in primary care settings (Bae et al., 2019; Gunal, 2012; Günal & Pidd, 2010; S. C. Brailsford et al., 2009). Very few studies have modelled the interaction between acute and community-based care (Onen Dumlu et al., 2022).

Agent-based simulation and discrete simulation are two approaches frequently used for modelling patient flows and bed capacity requirements. Agent-based simulation models the actions and interactions of autonomous agents within a system, to assess their effects on the system, over time. Agents may be individuals (e.g., patients or healthcare personnel), households, organisations, or even health care resources. Corsini et al. utilised agent-based modelling to simulate the impact of reconfigured pathways on patient flows through an oncology department, system efficiency and the time patients spent waiting (Corsini et al., 2022). When determining when to use different types of simulation, agent-based simulation “… may be particularly useful in modelling systems where the decisions of, and interactions between, individual agents and their actions are likely to affect those aspects of overall system behaviour under study”.

Discrete simulation models represent systems where changes in the state of the model occur at distinct points in time (Thierry et al., 2008). For example, when a patient arrives at an emergency room, the state of the emergency room – defined as the total number of patients present – changes. Discrete simulation is often used due to its flexibility and its intuitive outputs that are typically well understood by policymakers and those without a modelling background. Discrete simulations can be used to represent complex systems and behaviours occurring within and between individuals, populations, and their environments (Günal & Pidd, 2010; Karnon et al., 2012; Ramwadhdoebe et al., 2009; Robinson, 2014b; Zhang, 2018). There are two main approaches to making time advance in discrete simulation – from one event to the next, also known as discrete event simulation or in fixed increments, known as discrete time simulation (Chiang et al., 2020; Phillips, 2007; Robinson, 2014a; Thierry et al., 2008).

We identified several examples of discrete simulation modelling and hybrid approaches focusing on comparing care pathways for elderly patients to avoid hospital admissions and to facilitate discharge from hospitals (Katsaliaki et al., 2005; Ragab et al., 2013). Bruzzi et al. highlight the potential of using simulation techniques to explore different aspects of care processes for frail elderly patients requiring acute care (Bruzzi et al., 2018). Bae et al. is one of the few studies using discrete event simulation to model the relationship between supply and demand for long-term care beds in a large-scale provider network, to support long-term capacity planning (Bae et al., 2019). A recent study from the UK used discrete time simulation to estimate the appropriate capacity requirement for intermediate “step-down” care services within a healthcare system, during the COVID-19 recovery period (Onen Dumlu et al., 2022).

We were not, however, able to identify studies using simulation to model the flow of patients through MAUs or similar units. The lack of studies is not surprising given that access to MAU beds has only been mandatory in Norway since 2016. We did not identify simulation studies for similar units either. This may be because MAUs only target patients on their way into hospitals (acute step-up) but not on the way out (step-down). This is unusual in the international context where units are either used for both purposes or just for step down. The evidence-base for MAUs and MAU-like units is thus limited (Forsetlund et al., 2014; National Audit Office, 2016).

When identifying a suitable approach to modelling MAU occupancy, we had to consider that the numbers of MAU beds and MAU patients were relatively low. We also needed a model that could represent random arrivals and discharges as well as individual beds and patients, meaning that a systems dynamics model was not appropriate. We chose a simulation rather than a queueing model to better accommodate day of the week and seasonal variability in admissions. A stochastic simulation model was deemed most appropriate, allowing us to represent discrete arrival and discharge events.

Our intention was to create a reasonable representation of the system in the simplest way possible. We chose discrete time simulation rather than discrete event simulation as we only had daily numbers of arrivals and discharges but no arrival or discharge times. It thus made sense to represent the model time in steps of 24 h. This simplified the implementation, as all events were drawn at midnight each day and thus the usual discrete event mechanism of jumping to the next time point when an event occurs was not required. However, discrete time simulation requires aggregation of multiple events during a fixed time period, which results in the loss of some interactions between events thus making it less accurate than discrete event simulation (Chiang et al., 2020).

We also needed to identify a method for estimating the unconstrained demand for MAU beds, as we lacked historical data on the number of patients turned away because units were full. This is not an approach that has been regularly applied to healthcare settings but has been used in airline revenue management (Guo et al., 2012).

The aim of the study was to develop a discrete time simulation model to estimate the impact of different organisational scenarios on MAU occupancy. We validated our model using empirical MAU data and an analytical queueing model. In addition, we identified a method for indirectly estimating the number of patients turned away due to capacity constraints.

## 2. Materials and methods

We carried out a simulation study of patient flows through MAUs. Our simulation model was built in R. version 3.5.0 using the following packages: dplyr, lubridate, magrittr, broom, and ggplot2 (R Core Team, 2018).

The key policy goal for MAUs was based on the annual number of patient days – where a patient's day is defined as a day during which a person is confined to a bed and stays overnight in a hospital. In our study, however, the key performance indicator (KPI) for the MAUs was defined as their *absolute mean occupancy*. Absolute mean occupancy was defined as the expected value based on the average number of beds occupied at midnight over 365 days.

For the purposes of our analysis, the absolute mean occupancy was a more convenient measure to interpret than annual overnight stays, given its natural range from 0 to the MAU’s maximum bed capacity. An MAU’s expected contribution to annual patient days can be easily derived from absolute mean occupancy × 365.

### 2.1. Study setting

The study was based on patient data from 7,352 admissions to four MAUs located in south-east Norway in 2017. The MAUs were all located in the catchment area of a large university hospital. The MAUs varied in size, with the largest having 72 beds and the smallest having 6 beds. The characteristics of the individual study populations are presented in Table 1. MAU patients were typically older individuals over 70 years, with more females being admitted than men. The mean length of stay was just over 3 days with one notable exception (referred to as MAU 2 in the table below) which had a mean length of stay of 5.2 days.

**Table 1.** Study population characteristics.

MAU	All	1	2	3	4
Beds	107	14	6	72	15
Total number of admissions	7,352	1,061	168	5,243	880
Absolute mean occupancy (sd)	62.9 (10.1)	9.42 (2.55)	2.33 (1.35)	43.02 (7.68)	7.08 (2.43)
Median age (IQR)	82.0 (22)	76.0 (28)	79.5 (19)	83.0 (20)	78.0 (22)
Sex (%)					
Female	66.2	61.4	61.3	67.9	63.3
Mean length of stay in days (sd)	3.1 (2.3)	3.3 (2.7)	5.2 (4.2)	3.0 (2.1)	3.0 (2.3)

### 2.2. Simulation model

**Figure 1.**
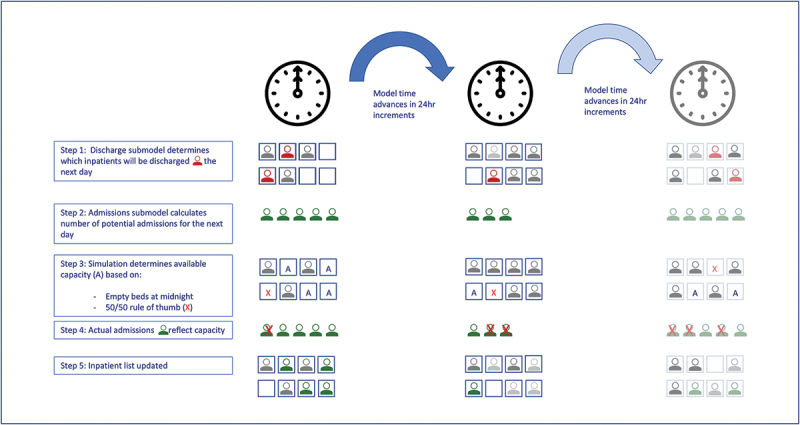
A graphical representation of the MAU discrete time simulation model.

We have uploaded the source code for our baseline simulation model to a github repository (https://github.com/tali-k/MAU-code). We provide a graphical representation of the model in Figure 1.

The simulation model maintained a list of inpatients for the MAU. It used a fixed increment approach with discrete time steps of 24 h and updated the patient list at each time step. At midnight each day, a discharge sub-model evaluated each inpatient and determined whether he/she would be discharged within the following 24-h period and taken off the list (Figure 1, step 1). The simulation model also included a sub-model for admissions, which generated a set of potential patients that could be admitted the following day (Figure 1, step 2). The number of potential patients that ended up being admitted (Figure 1, step 4) and thus added to the inpatient list (Figure 1, step 5), depended on the number of available beds (Figure 1, step 3).

Determining how to apply a capacity limit was not immediately obvious as the model computed discharges and potential new admissions at the beginning of the 24-h period. In reality, discharges and admissions take place at random times throughout the day. This means that some empty beds resulting from discharges will be available for use by new admissions on the same day but not always. To account for this, we implemented a 50–50 rule i.e., a bed vacated by a discharged patient had a 50% probability of being available that day for a new admission.

We used the model to estimate the absolute mean occupancy when there were no constraints on the number of beds available, which we referred to as the MAU’s potential occupancy.

The simulation model was also programmed to allow for the merging of MAUs, by simulating admissions and discharges in parallel, whilst pooling their bed capacity. This feature was used to estimate the benefits of merging MAUs on absolute mean occupancy.

#### 2.2.1. Sub-model for potential admissions

The sub-model for potential admissions was implemented as a two-step process. In the first step, a linear regression model estimated the expected number of potential admissions, and this number was passed on the Poisson function. A random integer number of patients was thus generated from a Poisson distribution whose mean was equal to the regression output. In the second step, the sub-model generated the same number of patients equivalent to the integer value from the Poisson distribution.

To carry out the first step, we estimated separate sets of regression coefficients for each MAU, using empirical data. Further details are given in Appendix A. For the regression output to reflect potential (rather than actual) admissions and thus unconstrained demand, we selected a subset of the empirical data set to include on “low occupancy” days where bed capacity was freely available. Low occupancy was defined as less than or equal to the median occupancy for a given MAU. This allowed us to apply a consistent approach across all MAUs and to still ensure that sufficient data points were available for our regression analyses.

The regression model used multivariate linear regression with day of week and month as categorical predictor variables. The variables included were based on the findings of a previous regression analysis of factors associated with the number of admissions to MAUs (Kakad et al., 2020). Absolute hospital occupancy was not associated with the number of MAU admissions and was thus not included in our model. MAU occupancy, weekday and month of admission were found to be significantly associated with the daily number of MAU admissions and thus were included. We chose not to include MAU occupancy as a variable in our admissions model, as the associated coefficients would only be valid for certain bed capacity and some of our experiments required us to alter MAU bed capacity. In addition, by selecting a subset of days where the occupancy was low, we could eliminate the effect of MAU occupancy on the number of admissions. It therefore made little sense to estimate this very effect by including occupancy as a predictor in our model.

#### 2.2.2. Sub-model for discharge

The discharge sub-model was implemented as a logistic regression model, as this is a standard way of modelling probabilities. We estimated separate sets of regression coefficients for each MAU, calibrated from empirical data, but in this case, we did not filter data based on occupancy. The sub-model determined the probability of discharge the following day for each inpatient. This was implemented by drawing a uniform (0,1) variable and comparing the result to the regression model output. The final model included the categorical variable “day of week” as the sole predictor variable in the logistic regression model. Variables that were not included in the final discharge model were age, sex, a binary variable for whether the patient resides in the municipality in which the MAU was located, MAU occupancy (number of occupied beds at midnight each day) and length of stay to date. The regression coefficients generated with only day of week in the model predicted empirical absolute mean occupancy most closely. We chose to omit age and sex in our discharge sub-model as sampling age- and sex-specific discharge probability distributions added little compared to sampling the overall discharge probability distribution. We did not derive length of stay distributions from historical data as the main outcome indicator of the study, absolute mean occupancy, only depended on the number of beds that were occupied over time. As such, reproducing a length of stay distribution was not a priority, particularly as the chosen approach was both simpler and accurately reproduced the empirical distribution of MAU occupancy.

#### 2.2.3. Simulation model assumptions

Simplifying assumptions are necessary – as with most models. We assumed that the stated capacity of the MAU corresponded to its maximum capacity, i.e., units did not increase the number of beds during periods of high demand. We did not consider financial, legislative, or staffing constraints – though these would obviously be important considerations prior to an actual implementation.

### 2.3. Model data, input parameters, and outcomes

We used de-identified individual-level patient administrative data for all admissions occurring in 2017 for four MAUs in south-east Norway. Variables in the dataset included date of admission, date of discharge, age, sex, and municipality of residence. We excluded records where admission or discharge dates were missing as this was considered essential for our analyses. As MAUs are intended for use as short stay units we treated admissions with length of stay over 21 days as recording errors and excluded them.

Our simulation model input parameters included the regression coefficients estimated for each MAU from the admission and discharge regression models mentioned previously. We estimated coefficients for day of week (excluding our reference day – Monday) and for each month (excluding our reference month January) from each of the admission and discharge models. We also used the reported maximum bed capacity for each MAU. The output of each run of our simulation model was the absolute mean occupancy based on MAU occupancy averaged over 365 simulated days. This output variable was collated for all 100 runs and absolute mean occupancy and 95% confidence intervals derived from these 100 data points.

### 2.4. Model verification and validation

The model code was verified in detail by the co-author, FD, through a separate implementation of the same model. We compared simulated values for the absolute mean occupancy for each MAU with empirical data from 2017.

### 2.5. Experiments

Our baseline model was adapted to carry out two types of experiments. The first of which estimated the reduction in absolute mean occupancy (“loss”) from MAUs resulting from a lack of beds. The second experiment estimated the benefits of merging MAUs in two different ways: Firstly, we estimated the increase in absolute mean occupancy for the merged unit when compared to the sum of the mean occupancies for the individual MAUs. We then estimated the number of beds that could be removed from the merged unit, while still maintaining the same absolute mean occupancy as the sum of the individual units.

The results of our experiments were based on the mean values over 100 runs of the model. Each run simulated 365 days of arrivals and discharges for each MAU, giving us a total of 36,500 days-worth of simulated data for each MAU. We included a warm-up period of 2 weeks to ensure a steady state in occupancy was achieved and a cool-down period of 7 days to ensure that we could calculate lengths of stay for all patients admitted over the course of a year.

#### 2.5.1. Experiment 1: estimating the reduction in absolute mean occupancy due to capacity constraints

Historical data on the number of patients turned away from MAUs due to capacity constraints was not available. Even if MAUs had recorded the number of patients turned away due to lack of beds, this data would not capture otherwise eligible patients that were not referred because the referring doctor knew that the MAU was full. Our simulation model allowed us to estimate the total loss of patients and hence the total impact on absolute mean occupancy. In experiment 1, we modelled potential admissions in the same way as the baseline model but removed the constraint on the maximum number of beds in use per day.

#### 2.5.2. Experiment 2: a) Merging MAUs without reducing the number of beds and b) Merging MAUs and minimising the total number of beds without affecting service provision

Our second experiment was divided into two parts. In the first part (2a), we simulated the effect of merging two or more MAUs on absolute mean occupancy. The number of beds in the merged MAU was equal to the sum of the individual MAUs. In the second part (2b), we estimated the number of beds that could be removed without affecting service levels. Service level was expressed as the combined absolute mean occupancy, where absolute mean occupancy values for the individual MAUs were those derived from the baseline model, table 2). These two experiments were carried out using our baseline model, ensuring that discharge model coefficients for the MAU from which the admission originated were applied when generating the daily probability of discharge for each inpatient. As soon as the capacity of the merged unit was exceeded, any remaining potential admissions were excluded from the model. This was done at random, to ensure that potential admissions coming from different MAUs had the same probability of being turned away due to capacity constraints.

**Table 2.** Baseline model validation.

MAU	Bed capacity	Empirical		Baseline (based on 100 runs of 365 days of the model)	
		Absolute mean occupancy	Standard deviation	Absolute mean occupancy (95% CI)	Standard deviation
1	14	9.42	2.55	9.27 (9.22–9.32)	0.26
2	6	2.33	1.35	2.57 (2.54–2.61)	0.19
3	72	43.02	7.68	43.33(43.19–43.48)	0.73
4	15	7.08	2.43	7.41 (7.35–7.47)	0.30

The three scenarios we chose each represent a distinct merging strategy. The first two scenarios were based around MAU 4, a medium-sized MAU with low absolute mean occupancy levels. In the first scenario, we merged MAU 4 with a smaller MAU 2 located in a similar geographic area, and thus might represent a more acceptable merger to patients residing locally than if MAU 4 was merged with a unit farther away. In the second scenario, MAU 4 was merged with the MAU with the highest occupancy (MAU 1) in the hope that the available capacity at MAU 4 might reduce the number of patients that might otherwise be turned away from MAU 1. In the third scenario, we explored gains in absolute mean occupancy obtained by merging all four MAUs. We used the same scenarios in the second part of the experiment, though the focus was on maximising the number of beds that could be repurposed without affecting service levels.

#### 2.5.3. Comparison with analytical model

We compared the results for the absolute mean occupancy from experiment 1 to the empirical values for each MAU and to the results of an analytical queueing model known as the Erlang loss model, which has been used previously for national-level analyses of MAU occupancies (28).

In the terminology of queueing theory, what we refer to as potential absolute mean occupancy (i.e., absolute mean occupancy in the absence of capacity constraints) is called the system’s load (symbolised by the Greek letter ρ). This formula gives the so-called loss (B) as a function of the bed capacity (n) and the load (ρ): 

(1) $$B\left(n,\rho \right)=\frac{\rho^{n}/n!}{\sum_{i=0}^{n}\rho^{i}/i!}$$

It represents the proportion of potential admissions that will not be realised because the unit is full. In most situations where such a model is applied the capacity and load would be given, and the task would be to use the estimated loss to calculate the occupancy:

(2) $$occupancy=\rho \left(1-B\left(n,\rho \right)\right)$$

However, for our application of the Erlang loss model the occupancy is the known variable -whilst the loss is the unknown variable. We need to find the load (potential occupancy) that is compatible with the empirical absolute mean occupancy and the MAU’s bed capacity. Equation (2) is highly non-linear in ρ, and we did not attempt to solve it analytically. Instead, we implemented a simple numerical scheme known as the bisection algorithm, which finds approximate solutions through a binary search on intervals.

## 3. Results

As previously mentioned, our admissions dataset used to estimate regression coefficients only included days where occupancy was less than or equal to the empirical median occupancy for the corresponding MAU. The proportion of days excluded (of the 365 days included in the original dataset) varied for each MAU, excluding the least data from MAU 1 (35%) and the most for MAU 3 (47%).

In Table 2 we compare the results of our baseline model with our empirical data for 2017. We see that our simulation model overestimates the absolute mean occupancy for MAUs 2 by 10,3% and by 4.7% for MAU 4, when compared to empirical data. For MAUs 1 and 3 our model estimates closely resemble empirical data.

### 3.1. Experiments

In the following section, we present the results from three experiments.

#### 3.1.1. Experiment 1

In Table 3 we show that when compared to empirical values, absolute mean occupancy increases for all MAUs when capacity constraints are removed (experiment 1). In absolute terms, the effect is most marked for MAU 1 where it appears that capacity constraints result in a reduction in mean daily occupancy by almost one patient (0.95). In relative terms, MAU 2 appeared most affected by capacity constraints, with empirical occupancy levels almost a fifth (19%) lower than our modelled estimates for unconstrained demand. Our estimates implied that MAUs 3 and 4 were least affected by capacity constraints, with percentage occupancy only increasing by 3% and 7%, respectively. As an additional check, we used an Erlang loss model to estimate absolute mean occupancy when for a given average demand and capacity. We once again observed that MAU 1 experienced the largest absolute increase. MAUs 2, 3, and 4 experienced no or only minimal increase in absolute mean occupancy.

**Table 3.** Comparison of absolute mean occupancy values from experiment 1 to results from the Erlang loss model and empirical data.

MAU	Absolute mean occupancy/load		
	Experiment 1*	Erlang	Empirical
1	10.4 (10.32–10.48)	9.98	9.42
2	2.76 (2.71–2.81)	2.39	2.33
3	44.2 (44.0–44.37)	43.02	43.02
4	7.52 (7.45–7.58)	7.11	7.08

* 95% Confidence Intervals.

#### 3.1.2. Experiment 2a

In Table 4 we have summarised the results of three different merging scenarios looking at the number of patient days generated. Merging increased absolute mean occupancy in all scenarios though the increase is greatest when we merge all four MAUs together (scenario 3).

**Table 4.** Combined results from experiments 2a and 2b.

Scenario	Merged MAUS	Combined number of beds	Increase in absolute mean occupancy+ (expt. 2a)	No. of beds that could be repurposed* (expt. 2b)
1	1+ 4	29	0.94 (0.81–1.07)	4
2	2 +4	21	0.25 (0.15–0.35)	2
3	1+ 2 + 3 + 4	107	2.09 (1.83–2.35)	20

+difference between simulated estimate from experiment 2 and the combined occupancy estimate from the baseline model (Table 2).

*simulation model estimates.

#### 3.1.3. Experiment 2b

In the second half of the merging experiment, we examined the same three scenarios to determine the number of MAU beds that could be repurposed while maintaining the existing level of service provision. Beds could be repurposed in all three scenarios – ranging from 2 (scenario 2) to 20 (scenario 3) beds – representing a 10% and 19% decrease in the number of beds, respectively.

## 4. Discussion

Providing community-based alternatives to hospital admissions is a priority for many countries. In Norway, over 200 MAUs have been established with this purpose in mind (Norwegian Directorate of Health, 2013; Norwegian Ministry of Health and Care Services, 2009; 2015).

MAUs have faced criticism for consistently low mean occupancy levels (Norwegian Directorate of Health, 2020b; Skinner, 2015b). Given that MAUs are well established and popular amongst MAU patients and their families alike, we believed that policymakers would prefer interventions aimed at improving the use of MAU beds rather than abandoning the concept of MAUs all together (Leonardsen, Grøndahl, et al., 2017). We therefore used a variety of modelling techniques, including simulation, analytical models, and regression modelling, to estimate the impacts of organisational interventions aimed at increasing MAU absolute mean occupancy.

We estimated the number of potential admissions to MAUs in our study in the absence of any capacity constraints. We did this in what we believe to be a robust and replicable manner, successfully combining the use of regression modelling and discrete event modelling. Our arrival model uses a Poisson function to generate arrivals. We considered this appropriate as arrival processes generally tend to be Poisson distributed since they are the result of many independent, low probability random events in a population (Green, 2013).

As we did not possess time-stamped data for admissions and discharges (only the date), we chose overnight stays as our preferred metric and discrete time simulation or “time buckets” rather than discrete event simulation. The lack of time-stamped data also necessitated some pragmatic model assumptions. For example, we include an assumption that a bed vacated by a discharged patient had a 50% probability of being available that day for a new admission. Whilst it was not possible to directly validate this assumption using data, we argue that the overall estimates of mean occupancies are in line with the historical data, providing indirect validation.

Our estimates suggest that in 2017 only MAU 1 exhibited substantial losses due to capacity constraints. Whilst this is positive news for local patients who are unlikely to be turned away, this finding could also be interpreted as a mismatch between the demand for and the supply of MAU beds. This is consistent with concerns raised early on that demand for MAU beds had been overestimated by the Directorate of Health (Bruun Wyller, 2014; Deloitte, 2015; National Audit Office, 2016; Skinner, 2015a). There have also been concerns that MAUs do not function as a substitute for hospital admission as per the original policy intention (Kakad et al., 2020; Leonardsen et al., 2016).

It is possible that patients being admitted to MAUs would not have been admitted anywhere had the MAU not been in place – alternatively MAUs could be providing a service that meets a previously unmet need (Skinner, 2015a, 2015b). In either case, it is not clear how one might increase the demand for MAU beds without considering more flexible use of beds or by expanding the criteria for admission. However, this was beyond the scope of our study.

The loss of patients due to capacity constraints is often difficult to quantify, and empirical data on turn-away rates was not available directly from MAUs. Even if MAUs were capturing the number of patients turned away, they would have no means of identifying the number of patients suitable for MAU that were never referred. This could happen in situations if the referring doctor was aware of capacity issues at the MAU, thus choosing an alternative course of management. By comparing simulated values for absolute mean occupancy with empirical data, we were able to quantify the impact that potential admissions lost due to capacity constraints have on absolute mean occupancy. We consider this to be a useful approach for obtaining a metric that is rarely captured in healthcare.

We triangulated our simulation estimates of absolute mean occupancy in the absence of capacity constraints with the results generated by a theoretical queueing model, the Erlang loss model. This model is much simpler in its construction and data requirements but allows us to indirectly estimate the load for a given empirical absolute mean occupancy and bed capacity. The losses generated by the Erlang model were considerably lower than those from our simulation of potential admissions in the absence of capacity constraints (experiment 1). This could be expected as the Erlang model assumes a steady Poisson rate of admissions, meaning the probability of a patient being admitting at a given point in time is independent of the time interval since the previous patient was admitted. This assumption is not unreasonable as emergency arrivals typically follow a Poisson distribution, however the rate of admissions in real life (and in our simulation models) tends to exhibit day of week and monthly variation (Green, 2013). This means that in real life, we would expect a greater number of patients to be turned away than the overly optimistic estimates of an Erlang model – a phenomenon referred to elsewhere (Kakad et al., 2019). As such, we consider this justification that our more detailed simulation approach was appropriate in this case.

We simulated the effects of merging MAUs to show the extent to which merging satisfies the true demand for those MAU beds. As one might expect from the low levels of capacity constraint identified in experiment 1, merging units resulted only in a modest increase in absolute mean occupancy in all scenarios. The greatest relative increase in occupancy was for scenario 1 where we merged two similar sized units – one with lower occupancy and the other with a degree of capacity constraints. The greater absolute increase resulted from merging all four MAUs.

We also attempted to quantify the number of MAU beds that could be removed or repurposed for alternative use – for example, as step-down beds for patients at the local hospital or short-term social care beds – while still maintaining absolute mean occupancy levels. We found that we could reduce the numbers of beds in all three scenarios. If all four units were merged 20% of existing beds could be removed or repurposed. Whilst there may be practical and political reasons for not centralising MAUs, we considered it important for policymakers to understand the extent to which centralising MAU may reduce the need for beds and free up resources that may be used elsewhere.

Merging MAUs would require consideration of factors such as changes in mean travel times for patients and where a physical merger was suggested to determine whether available space and staffing was available. Virtual mergers (if an MAU is full, it may route suitable patients to a partner MAU with available capacity) may result in less upheaval and more effective usage of empty beds but may not provide economies of scale in terms of reducing fixed costs related to buildings or equipment that would still be required at both sites.

### 4.1. Strengths and weaknesses

Our study has two main strengths. The first is that we provided insights relevant to healthcare policy and decisionmakers in Norway and elsewhere. We successfully combined different modelling approaches in a robust manner, that we believe can be generalised to other health policy or operational questions – particularly those pertaining to MAUs and similar admission avoidance units. We are not aware of other studies that have modelled admissions and discharges to MAUs nor of other examples of studies that have assessed the operational impacts of MAU specific policies. Time bucket modelling and other stochastic approaches, however, have been used to answer similar questions in supply chain modelling e.g., in warehouse management, where discrete event simulation may be computationally too costly or where a simpler approach is preferable (Chiang et al., 2020; Gong & de Koster, 2011; Thierry et al., 2008).

The second main strength is that we presented an approach to a common challenge faced by modellers. Modellers often lack data on the number of patients that are turned away due to capacity constraints, which impact MAU. This makes it difficult to accurately estimate unconstrained demand for beds using historical data. We described a simple means of indirectly estimating unconstrained demand.

We favoured the use of R rather than proprietary simulation software as it was open source. As our model did not require patients to compete and queue for resources, the in-built calendar-based registers of pending events common to proprietary simulation software were not useful in this context. In addition, we would have had to program essential features of our simulation model irrespective of whether we used proprietary software or R. There are R packages available for discrete event simulation and time slice modelling, but these were not easily adapted to our purposes.

The population of the MAUs included in the study covered a large geographic area, predominantly urban with some rural areas. As such, we would expect the population to be reasonably representative for Norway. In terms of generalisability of findings, the MAUs included in the study were larger than the national average, which is below three beds (Norwegian Directorate of Health, n.d.). As smaller units are more vulnerable to variability of patient flows, they are more likely to turn patients away due to capacity constraints. However, given their small size, this translates to relatively few patient days lost per MAU in practice. Whilst there may be a question of temporal generalisability, we believe 2017 to be a representative period of activity as each of the study MAUs was operating at its current capacity and had been operational for at least 1 year if not more.

We estimated our admissions coefficients for our simulation model using a subset of data to mimic a situation where capacity was unconstrained. This approach worked for the MAUs in our study where the median occupancy was well below the maximum capacity. This would not have been appropriate in a highly congested system where median occupancy was at or near full capacity. We required sufficient data to estimate the parameters accurately. By using the median approach, we were able to use the same approach for all MAUs whilst still retaining adequate amounts of data for each of them.

### 4.2. Policy implications and future research

Merging MAUs may offer some efficiency gains and improvements in bed utilisation. However, our findings suggest it is unlikely that mergers as a single intervention would substantially relieve pressures on hospitals. This could be explained if the types of patients admitted to MAUs belonged to a group that would otherwise have never been admitted to hospital. The “diagnostic loop” routes potentially suitable patients via acute medical admissions units at local hospitals to screen their suitability for MAU admission. Whilst theoretically ensuring that the appropriate patients are admitted to MAUs, this approach adds an extra step and potential delays to the patient pathway. This loop has been implemented for certain MAUs but requires formal evaluation to determine its effect. Another topic for future discussion with policymakers and healthcare providers is whether admission criteria for MAUs should be expanded. For example, MAU beds could be for step-down purposes for patients recently discharged from hospital.

Our study only looked at beds but no other types of essential resources (such as numbers and types of staff), which might limit the effect of merging MAUs on bed utilisation. Future modelling efforts could attempt to quantify the changes in resource requirements that result from merging MAUs. In addition, estimates of mean distances from patients’ homes would be a useful parameter to assess, as merging units would likely result in mean travel times or distances increasing. Determining the optimal location of MAUs for a given population could also be an interesting topic for future research. The models could inform discussions regarding trade-offs between distance from home, bed occupancy levels, and even patient satisfaction – assuming the data are available.

Given the diverse nature and labelling of intermediate care facilities worldwide and in the literature, we think an authoritative, systematic review that delineates intermediate care initiatives and reviews associated modelling literature would be valuable.

Our methodological approach is of interest to policymakers given its generalisability to other types of health care problems – particularly when capacity planning, trying to determine capacity constraints or assessing the impact of organisational change.

## 5. Conclusion

Our work provides insights for Norwegian policymakers and has relevance for step-up units in general – particularly those located in sparsely populated regions facing low occupancy levels. It also describes a methodological approach for estimating the demand for a service in the absence of capacity constraints – using model inputs derived from systems with capacity constraints.

Our study suggests that some increase in occupancy can be achieved by merging units – though the gains are relatively modest given that units being merged rarely achieved full capacity anyway. Substantially increasing the productivity of small units such as MAUs without changing admission criteria and the intensity of care offered is likely to be challenging.

Our results also suggest that, based on 2017 data, there appears to be scope for removing or repurposing beds without affecting existing service levels. Any attempts to repurpose beds should consider and mitigate against adverse impacts on MAU patients and avoid further fragmentation or increased logistical complexity of care pathways for alternative patient groups.

## Methods

*Settings*

We used data from four MAUs located in the south-east of Norway. All four units belonged to a single hospital catchment area. The hospital was university hospital serving a population of over 500 000. In 2017, there were five other MAUs also serving the population in the catchment area for Hospital 1 which were not included in our study. Of these five MAUs, three were considered out of scope as they primarily served other hospital catchment areas with only a minority of patients belonging to Hospital 1. One was removed as analysis of empirical data implied that the stated maximum number of beds during our study period was not applied consistently. The remaining MAU did not respond to our request to participate.

### Study period

Data were available for different time periods for each MAU. The study period of 1 January 2017 until 31 December 2017 was the period for which data were available across all MAUs and bed capacity remained constant at each MAU.

### Inclusion and exclusion criteria

Only admissions for patients aged 18 or over were included in the study. Records where age or sex or admission or discharge dates were not recorded were excluded. Same-day discharges were excluded from discharge and admissions analyses. We have included all 2017 events for both discharge and admissions. For those admitted in 2016 and discharged in 2017, only discharge events were included in the analyses. Similarly, for those admitted in 2017 and discharged in 2018 only admissions events were included. Admissions with lengths of stay over 21 days were determined as likely recording error and excluded. A detailed overview can be found in appendix A, table A1.

### Data for admission model

Coefficients for the admission model were estimated using a subset of admissions data, including only days where MAU absolute occupancy (i.e., the number of patients present at midnight at the start of the day) was equal to or greater than the median occupancy for the unit for 2017.

### Model coefficients

Admissions model

**Table:**

*Dependent variable: Admissions*				
*Linear (OLS)*				
MAU	1	2	3	4
Intercept	1.80*** (0.91, 2.69)	0.51** (0.05, 0.97)	20.63*** (13.49, 27.76)	2.06*** (0.69, 3.42)
Weekday Monday	0.49 (−0.37, 1.36)	0.40** (0.03, 0.76)	1.31 (−0.97, 3.59)	0.79* (−0.01, 1.59)
Weekday Tuesday	−0.38 (−1.20, 0.44)	0.06 (−0.30, 0.42)	1.38 (−0.64, 3.41)	0.29 (−0.49, 1.07)
Weekday Wednesday	0.68* (−0.06, 1.42)	−0.09 (−0.45, 0.27)	0.91 (−0.97, 2.78)	0.06 (−0.72, 0.84)
Weekday Thursday	0.28 (−0.51, 1.06)	0.09 (−0.26, 0.43)	1.21 (−0.57, 3.00)	0.25 (−0.49, 0.99)
Weekday Friday	0.34 (−0.42, 1.09)	0.16 (−0.19, 0.51)	2.37*** (0.62, 4.13)	0.54 (−0.22, 1.30)
Weekday Saturday	−0.50 (−1.27, 0.27)	−0.10 (−0.44, 0.25)	−0.12 (−1.88, 1.63)	0.39 (−0.31, 1.09)
Month February	0.82 (−0.31, 1.95)	−0.31 (−0.89, 0.26)	−7.37* (−15.00, 0.26)	0.15 (−1.34, 1.63)
Month March	1.19** (0.15, 2.23)	−0.35 (−0.82, 0.12)	−8.27** (−15.43, −1.11)	−0.29 (−1.78, 1.20)
Month April	1.26** (0.24, 2.29)	0.003 (−0.57, 0.57)	−9.26** (−16.44, −2.08)	−0.22 (−1.70, 1.26)
Month May	1.02* (−0.01, 2.05)	−0.35 (−0.84, 0.15)	−8.83** (−16.03, −1.64)	1.07 (−0.48, 2.63)
Month June	0.44 (−0.52, 1.41)	−0.21 (−0.69, 0.26)	−8.05** (−15.22, −0.88)	−0.60 (−2.07, 0.87)
Month July	0.84 (−0.21, 1.90)	0.04 (−0.53, 0.61)	−9.49** (−16.64, −2.34)	−0.24 (−1.72, 1.25)
Month August	0.47 (−0.46, 1.41)	0.05 (−0.48, 0.57)	−9.22** (−16.35, −2.10)	−0.38 (−1.86, 1.09)
Month September	0.75 (−0.27, 1.78)	−0.05 (−0.59, 0.49)	−8.43** (−15.61, −1.25)	−0.19 (−1.73, 1.34)
Month October	1.49*** (0.44, 2.53)	0.06 (−0.46, 0.57)	−9.09** (−16.24, −1.93)	−0.56 (−2.07, 0.94)
Month November	1.61*** (0.54, 2.68)	−0.11 (−0.63, 0.41)	−9.78*** (−17.02, −2.53)	−0.57 (−2.06, 0.92)
Month December	1.78*** (0.64, 2.92)	0.24 (−0.33, 0.81)	−9.82** (−17.47, −2.16)	−0.11 (−1.66, 1.44)
Observations	236	200	192	213
R^2^	0.14	0.12	0.13	0.10
Adjusted R^2^	0.07	0.03	0.05	0.02
Residual Std. Error	1.63 (df = 218)	0.68 (df = 182)	3.53 (df = 174)	1.50 (df = 195)
F Statistic	2.10*** (df = 17; 218)	1.41 (df = 17; 182)	1.55* (df = 17; 174)	1.29 (df = 17; 195)

*p<0.1; **p<0.05; ***p<0.01.

Discharge Model (logistic)

**Table:**

*Dependent variable: Probability of discharge*				
*Logistic*				
MAU	1	2	3	4
Constant	0.13*** (0.10, 0.17)	0.04*** (0.02, 0.10)	0.19*** (0.17, 0.21)	0.19*** (0.14, 0.25)
Weekday Monday	3.39*** (2.45, 4.75)	5.36*** (2.13, 16.40)	2.83*** (2.46, 3.25)	2.42*** (1.72, 3.44)
Weekday Tuesday	4.65*** (3.37, 6.50)	5.70*** (2.28, 17.34)	2.90*** (2.52, 3.34)	2.34*** (1.66, 3.33)
Weekday Wednesday	3.34*** (2.38, 4.73)	6.55*** (2.65, 19.81)	2.79*** (2.42, 3.22)	2.39*** (1.69, 3.40)
Weekday Thursday	3.39*** (2.43, 4.78)	5.88*** (2.33, 18.01)	2.77*** (2.40, 3.20)	2.19*** (1.54, 3.15)
Weekday Friday	5.00*** (3.61, 7.02)	10.41*** (4.27, 31.29)	3.35*** (2.91, 3.86)	4.10*** (2.92, 5.81)
Weekday Saturday	1.41* (0.97, 2.05)	1.56 (0.48, 5.43)	1.24*** (1.06, 1.45)	1.33 (0.90, 1.96)
Nagelkerke’s R2	0.07	0.09	0.05	0.05
Observations	3,439	849	15,703	2,586
Log Likelihood	−1,942.01	−374.89	−9,377.01	−1,522.52
Akaike Inf. Crit.	3,898.01	763.78	18,768.02	3,059.05

*p<0.1; **p<0.05; ***p<0.01.
